# Knowledge, Attitudes, and Practices of University Students Regarding the Impact of Smokeless Tobacco, Areca Nut, E-cigarette Use on Oral Health

**DOI:** 10.7759/cureus.66828

**Published:** 2024-08-13

**Authors:** Ankita Sarkar, Divya Vinayachandran, Ganesh C., Shanthi M., Sibyl Siluvai, Prashanthi Gurram, Lakshmi Rathan A. C., Mitthra S., Kalaivani V., Rajapandian K.

**Affiliations:** 1 Oral Medicine and Radiology, SRM Kattankulathur Dental College and Hospital, SRM Institute of Science and Technology (SRMIST), Chengalpattu, IND; 2 Public Health Dentistry, SRM Kattankulathur Dental College and Hospital, SRM Institute of Science and Technology (SRMIST), Chengalpattu, IND; 3 Oral and Maxillofacial Surgery, SRM Kattankulathur Dental College and Hospital, SRMIST, Chengalpattu, IND; 4 Oral and Maxillofacial Surgery, SRM Kattankulathur Dental College and Hospital, SRM Institute of Science and Technology (SRMIST), Chengalpattu, IND; 5 Conservative Dentistry and Endodontics, SRM Kattankulathur Dental College and Hospital, SRM Institute of Science and Technology (SRMIST), Chengalpattu, IND; 6 Periodontology, SRM Kattankulathur Dental College and Hospital, SRM Institute of Science and Technology (SRMIST), Chengalpattu, IND

**Keywords:** e-cigarette, areca-nut, smokeless tobacco, adolescence, university students, tobacco

## Abstract

Background: The prevalence of smokeless tobacco, areca nut, and e-cigarette use among university students has raised concerns regarding their potential impact on oral health. Assessing students' knowledge, attitudes, and practices toward these substances is necessary. Understanding these factors can inform targeted interventions and policies promoting oral health among this population. This study aims to investigate the awareness levels, attitudes, and behaviors of university students regarding smokeless tobacco, areca nut, and e-cigarette use and their potential effects on oral health. Identifying gaps in knowledge and misconceptions will help guide educational initiatives and public health interventions tailored to the needs of university students.

Methodology: The study employed a designed questionnaire, comprising 20 closed-ended queries, administered via the Google Forms platform. This survey was disseminated among college students in Chennai to gather insights for the study.

Results: Of the 500 college students included in the study, almost 85.5% (427) were aware of the harmful effects of smokeless tobacco on oral health. Additionally, 74.6% (373) recognized that chewing areca nut contributes to poor oral health, while 59.8% (299) acknowledged the negative impact of e-cigarette use.

Conclusions: The present study concluded that 11.1% (6) used smokeless tobacco, 18.9% (94) chewed areca nut, and 1.9% (10) used areca nut along with tobacco. Regarding e-cigarettes, 3.9% (19) indulged in vaping as a substitute for smoking regular cigarettes and 12.1% (61) used it along with tobacco cigarettes. It was also noted that 27.8% (139) were trying to quit and 12.6% (63) have tried but could not succeed. Therefore, although there was awareness of the ill effects of these deleterious habits on oral health and the increased risk of oral cancer, there remains a need to educate individuals and provide support for quitting these habits. Our study will provide insights into the present scenario of the awareness of the association between oral health and tobacco/areca nut consumption among college students in Chennai city.

## Introduction

Maintaining a radiant smile is not just about aesthetics; it is a reflection of overall health and well-being. Embracing a robust oral hygiene regimen is not merely a habit; it is a commitment to safeguarding yourself against a myriad of oral health concerns that could potentially compromise your general health and well-being [1].

Delving into the intricate web of oral health, it becomes apparent that tobacco, in its various forms, stands as a formidable adversary. The deleterious effects of tobacco on oral health are indisputable [2]. As per the Global Adult Tobacco Survey-India (2009), above one-third (34.6%) of Indians use tobacco in the form of smoking, chewing, application to teeth and gums, or sniffing. Tobacco and areca nut have been implicated in the development of oral potentially malignant disorders, which when left untreated proceed into malignancy [3]. Jose et al. reported a prevalence of oral potentially malignant disorders to be 13.28% among the rural population in South India [3]. Their study reported a prevalence of oral submucous fibrosis at 46.8%, leukoplakia at 28.4%, and erythroplakia at 9.7%.

In India, tobacco products proliferate at alarmingly low prices, perpetuating a cycle of addiction and despair [4].

Venturing further into the labyrinth of tobacco-related maladies, one encounters the pernicious specter of oral cancer, lurking ominously in the shadows. Smokeless tobacco products and betel quid, entrenched in the cultural fabric of India, emerge as notorious culprits in the proliferation of this dreaded disease [5]. Yet, despite its prevalence, oral cancer often remains shrouded in ignorance, its ominous presence concealed by a veil of apathy and misinformation, leading to tragic delays in diagnosis and treatment [6,7].

But the ravages of tobacco extend a lot. From cardiovascular maladies to respiratory afflictions, the collateral damage wrought by tobacco and its insidious cohorts knows no bounds [2,8,9].

Enter the e-cigarette, heralded as a beacon of hope in the war against tobacco addiction [10,11]. With its sleek design and promise of harm reduction, the allure of the e-cigarette knows no bounds, captivating the hearts and minds of a new generation. Yet, beneath its veneer of sophistication lies a dark underbelly of uncertainty. Research suggests that prolonged exposure to e-cigarette vapor can unleash a cascade of detrimental effects on the cardiovascular and respiratory systems, casting a pall over its purported benefits [12-15].

Despite the siren call of the e-cigarette, a veil of ignorance shrouds its true impact, obscuring the long-term consequences from the prying eyes of its users [10,16]. The habit of tobacco consumption has been shown to start in the adolescent age group and continue into adulthood. According to the World Health Organization, adolescents are most vulnerable to risk-taking behavior leading to substance abuse. In India, nearly 1 in 10 adolescents in the age group 13-15 years have ever smoked cigarettes. Tobacco is a risk factor for cardiovascular diseases, chronic obstructive pulmonary diseases, cancers, and oral diseases. Even after regulations against the use of these products, the second round of the Global Adult Tobacco Survey (2016-2017) revealed that 21.4% of adults in India consumed smokeless tobacco [3]. Thus, the imperative to educate and empower our youth, equipping them with the knowledge and tools to navigate the treacherous waters of tobacco addiction and oral health, becomes all the more pressing [2].

## Materials and methods

The cross-sectional study employed a self-administered, closed-ended questionnaire created on the Google form platform. This survey extending for a time frame of six months, consisting of 20 closed-ended questions, was distributed randomly to various colleges in and around Chennai. Institutional ethical clearance was obtained.

A validated questionnaire aiming to collect details from those college students who consented to participate by opening the Google link was circulated to obtain demographic data and details about smoking, smokeless tobacco use, areca nut (betel nut) chewing, and e-cigarette use (Appendix).

Sample size

All the college students who consent to be part of the survey (by clicking on the yes option which is given on opening the Google form link) will be involved.

The sample size was calculated by OpenEpi software, using the formula n= 4pq/d², and was estimated to be 500 according to a previously published article [1].

Quantitative assessment

The approach for the study involves inputting the collected data into Microsoft Excel, followed by transferring it to Statistical Package for the Social Sciences (SPSS) Version 25 (IBM Corp., Armonk, NY) for statistical analysis. Descriptive statistics, such as frequency and percentage, will be computed and displayed using tables and graphs. Variable comparison will be performed utilizing the Chi-square test.

The level of significance was set at 5%. A *P*-value < 0.05 was considered to be statistically significant.

## Results

**Figure 1:**
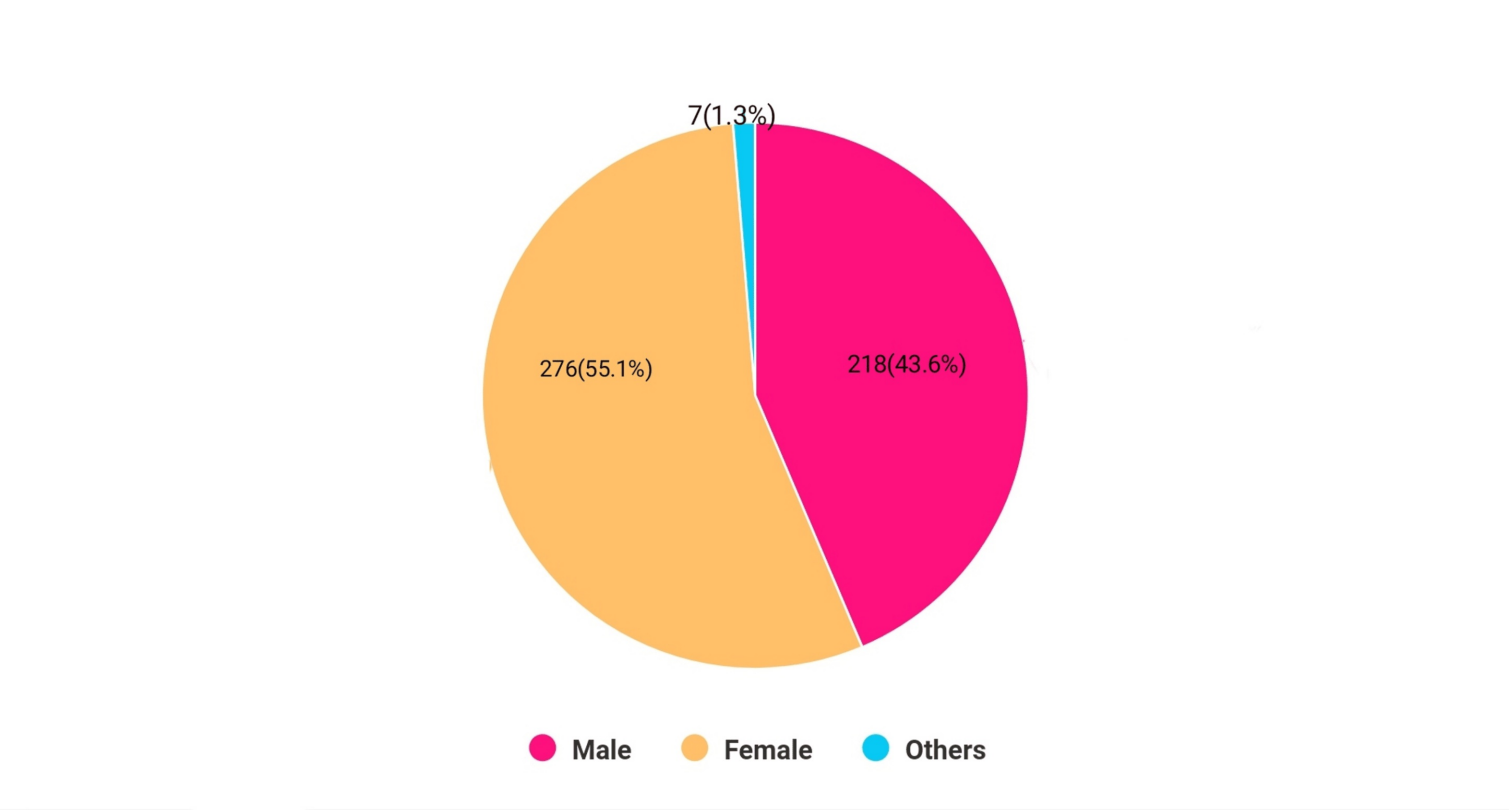
Gender distribution.

Out of a cohort of 500 students, the gender breakdown unveiled a striking narrative: 276 (55.1%) were female, 218 (43.6%) were male, and 7 (1.3%) identified as "other," defying conventional categorization (Figure 1).

**Figure 2:**
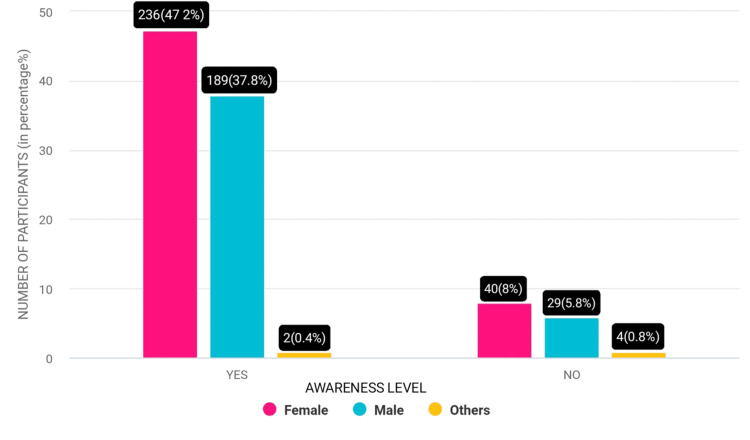
Awareness of smokeless tobacco impact on oral health among participants.

The awareness among females regarding the association between chewing smokeless tobacco and poor oral health stands at approximately 236 (47.2%), while for males, it was slightly lower at about 189 (37.8%). Interestingly, only a small proportion of males, approximately 29 (5.8%) participants, remained unaware that chewing smokeless tobacco can lead to poor oral health. This indicates a relatively higher level of awareness among young males within this demographic, although there is still room for improvement in educating them about the risks associated with smokeless tobacco use on oral health (Figure 2).

**Figure 3:**
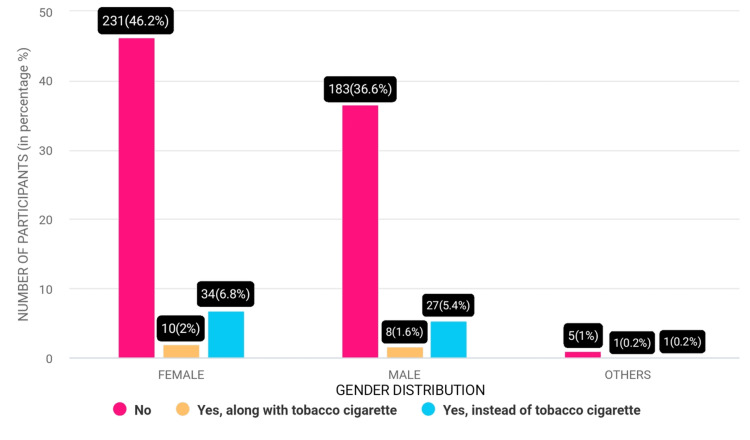
Usage of e-cigarettes among participants.

Among the participants, a vast majority, approximately 420 (84%), did not use e-cigarettes. However, a small percentage, around 19 (3.9%), opted for e-cigarettes instead of traditional tobacco cigarettes. Additionally, approximately 61 (12.1%) participants used e-cigarettes in conjunction with tobacco cigarettes, indicating a dual usage pattern among a notable minority (Figure 3).

**Figure 4:**
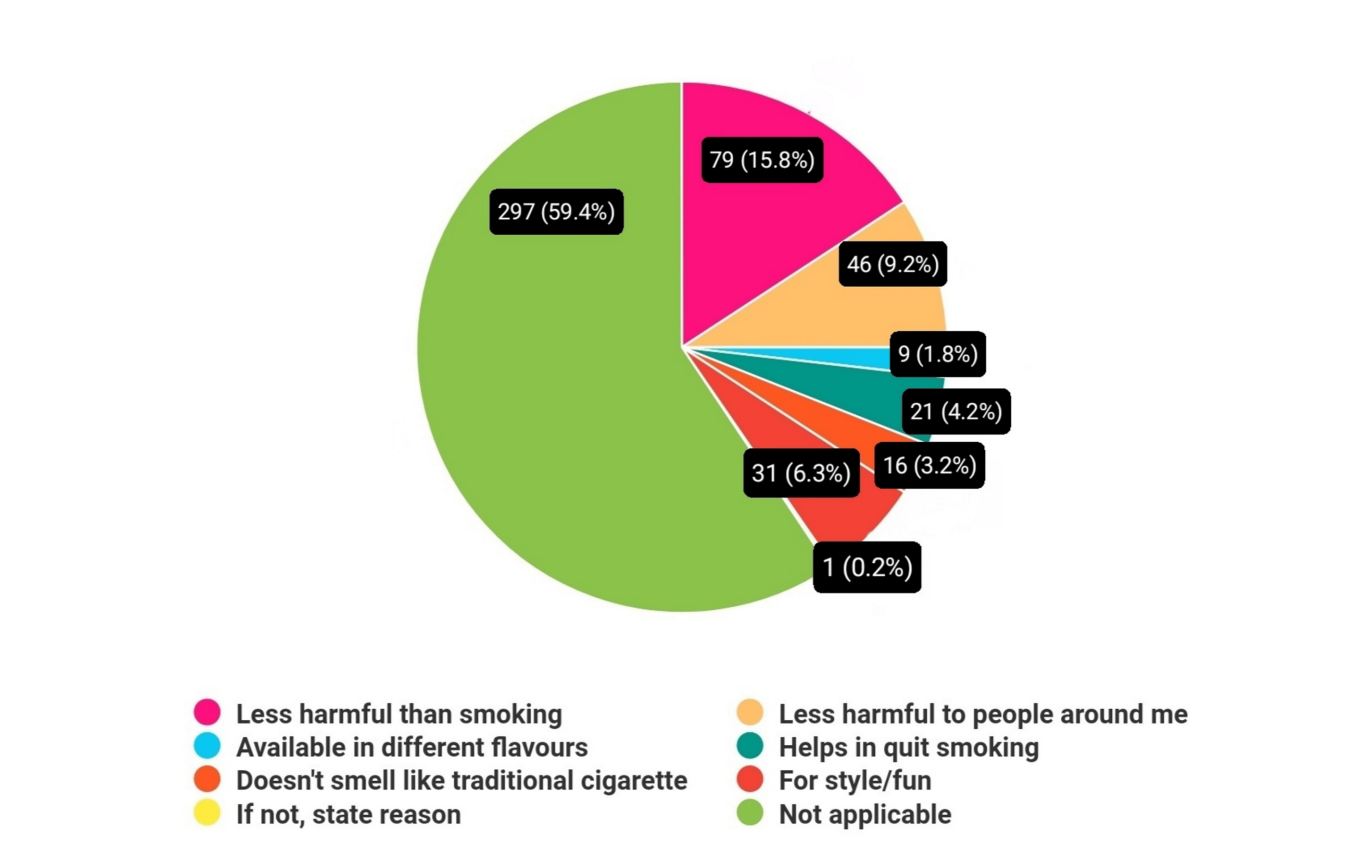
Reasons to use e-cigarettes/vaping among participants.

Among the participants who used e-cigarettes, a variety of reasons motivate their usage. Approximately 79 (15.8%) perceived e-cigarettes as less harmful than traditional smoking, while 46 (9.2%) believed it poses less risk to those around them. Another 9 (1.8%) were attracted to e-cigarettes due to the availability of diverse flavors, adding to the appeal. Interestingly, 21 (4.2%) viewed e-cigarettes as a tool to aid in smoking cessation, indicating a potential perception of harm reduction. Moreover, 16 (6.2%) appreciated the lack of smell associated with e-cigarettes compared to traditional smoking, enhancing the convenience factor. A negligible fraction, around 31 (6.2%), used e-cigarettes for recreational purposes or as a fashion statement, highlighting diverse motivations among users. Notably, the majority, comprising 297 (59.4%), did not use e-cigarettes at all, indicating a significant portion of nonusers within the surveyed population (Figure 4).

**Figure 5:**
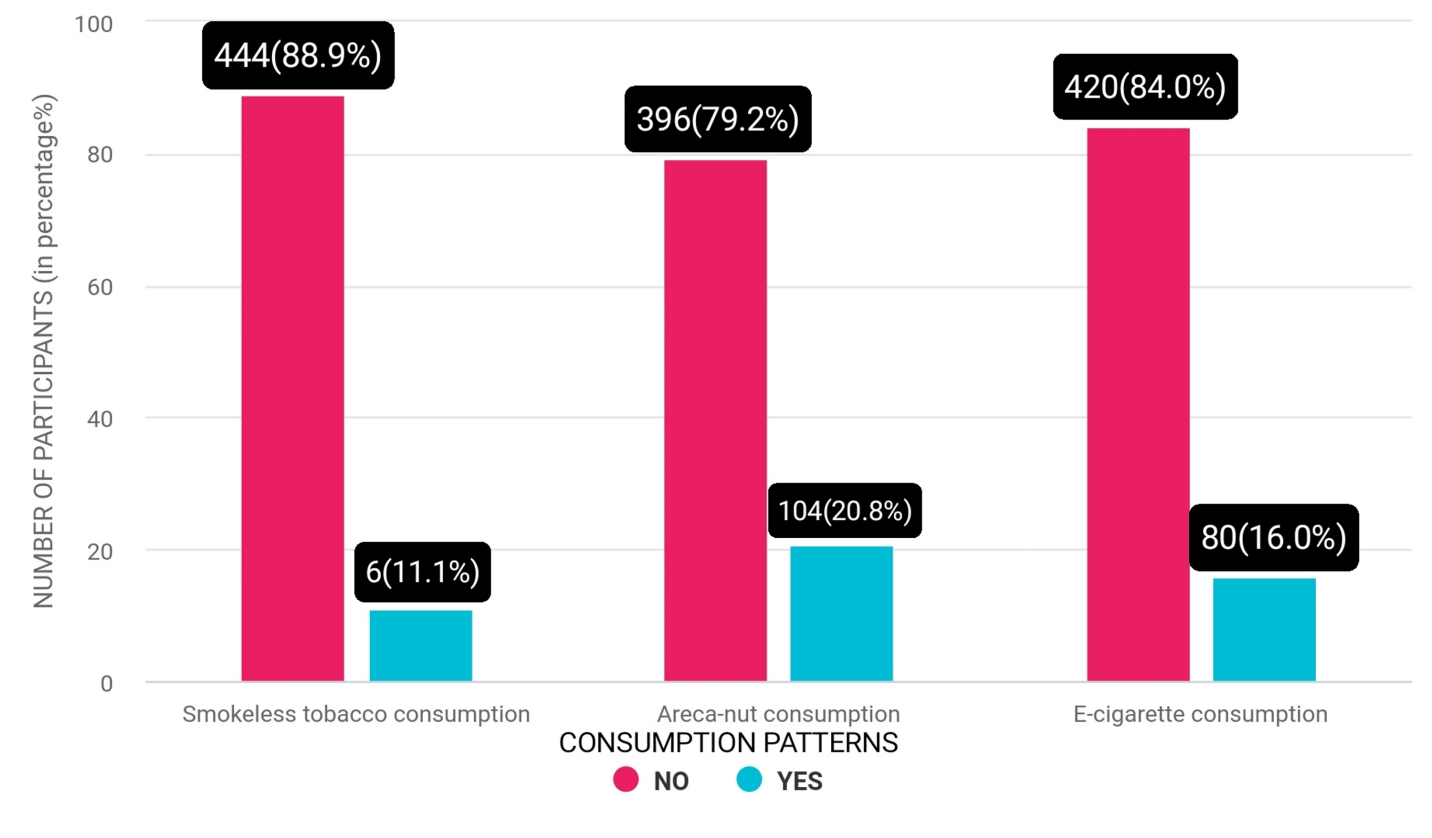
Consumption of smokeless tobacco areca nut and e-cigarette among participants.

The majority of participants had the habit of consuming areca nut, likely due to misconceptions and a lack of knowledge about its adverse effects. This may be influenced by traditions and cultural practices, as it is common in many parts of India to offer these commercial products to guests at social gatherings, weddings, and other cultural events (Figure 5).

**Figure 6:**
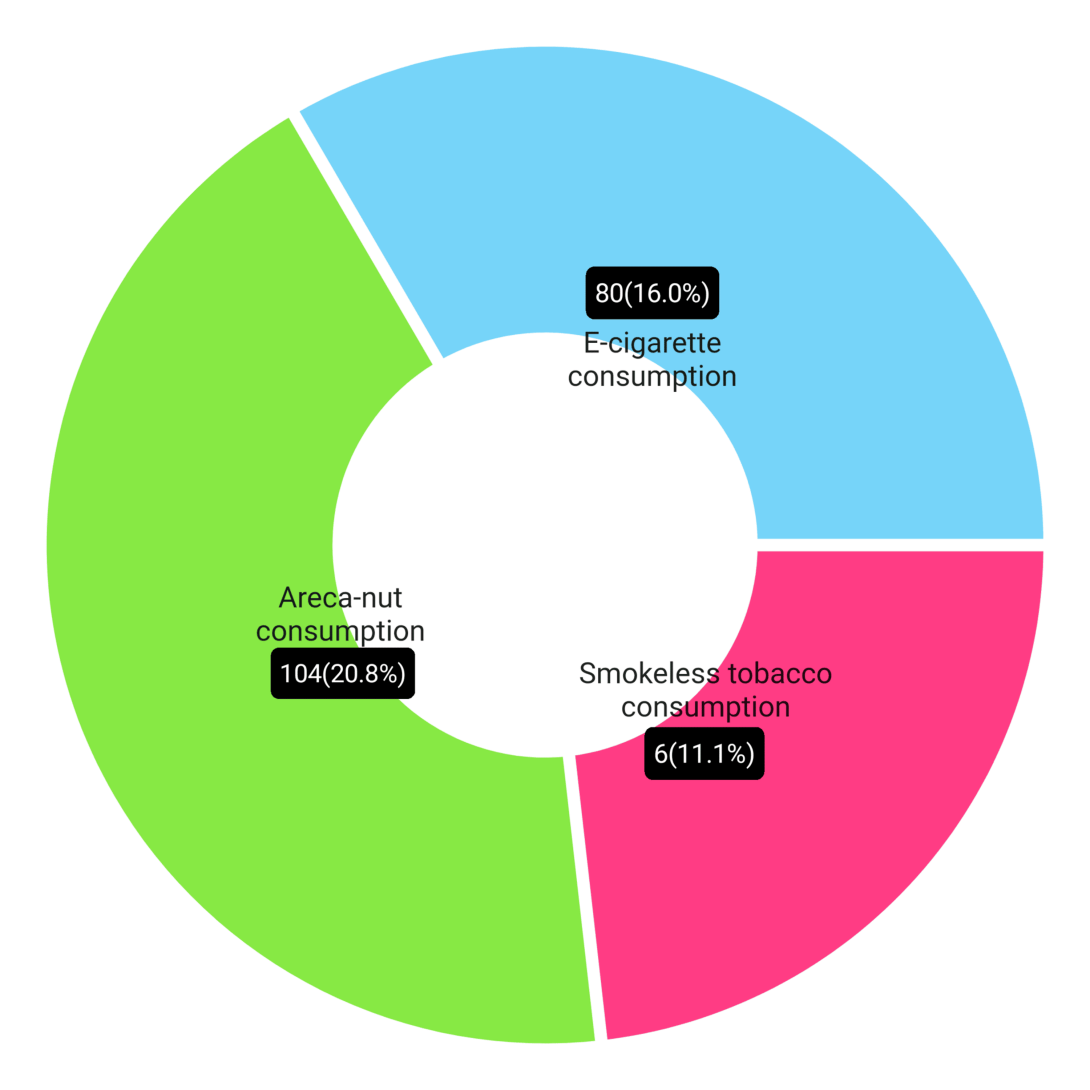
Comparison between the consumption of smokeless tobacco, areca nut, and e-cigarette.

Majority of the people used it as a mouth freshener, as 89 (17.9%) participants were not aware that mouth fresheners like Pan Parag and Manik Chand contain areca nuts. About 6 (11.1%) participants mentioned they chew tobacco. Ninety-four (18.9%) participants agreed to chew only areca nut, whereas 2 (1.9%) participants agreed that they chew areca nut along with tobacco (Figure 6).

E-cigarette is the sensation of our new generation. Most participants adopted e-cigarette use and vaping to follow a new trend. The majority of them picked up these habits from their friend circles. About 19 (3.9%) participants used e-cigarette instead of tobacco cigarettes, whereas 61 (12%) participants used tobacco cigarettes. There was a huge lack of awareness and misapprehension about the harmful effects of e-cigarette. About 249 (49.9%) participants believed that it is less harmful than tobacco cigarettes, whereas about 114 (22.8%) participants thought it is absolutely safe to use e-cigarette (Table 1).

**Table 1:** P-values of various questions. Chi-square test was used for the comparison. *Statistically significant (*P*-value ≤ 0.05). *n*, frequency; %, percentage

S. no.	Questions	Options	Responses of participants, *n* (%)	Chi-square	*P*-value
1.	Chewing smokeless tobacco (gutkha, khaini, and hans) leads to poor oral health.	Yes	427 (85.5%)	19.457	0.000*
		No	73 (14.5%)		
2.	Do you think that chewing smokeless tobacco leads to staining of teeth and gum diseases?	Strongly agree	198 (39.7%)	83.944	0.000*
		Agree	226 (45.3%)		
		Neither agree nor disagree	54 (10.8%)		
		Disagree	13 (2.6%)		
		Strongly disagree	9 (1.7%)		
3.	Do you think that chewing smokeless tobacco increases the risk of oral cancer?	Strongly agree	196 (39.1%)	95.958	0.000*
		Agree	222 (44.5%)		
		Neither agree nor disagree	62 (12.4%)		
		Disagree	12 (2.4%)		
		Strongly disagree	8 (1.5%)		
4.	Do you have the habit of chewing smokeless tobacco (gutkha, khaini, and hans)	Yes	6 (11.1%)	30.511	0.002*
		No	444 (88.9%)		
5.	Areca nut (also called betel nut) is present in commercial products like Pan Parag, Manik Chand pan masala, Ajantha supari, etc.	Yes	411 (82.1%)	6.122	0.805
		No	89 (17.9%)		
6.	Chewing areca nut (betel nut) leads to poor oral health.	Yes	373 (74.6%)	3.315	0.913
		No	127 (25.4%)		
7.	Do you think that chewing areca nut (betel nut) can lead to staining of teeth and gum diseases?	Strongly agree	169 (33.8%)	40.428	0.000*
		Agree	216 (43.2%)		
		Neither agree nor disagree	89 (17.8%)		
		Disagree	17 (3.3%)		
		Strongly disagree	9 (1.9%)		
8.	Do you think that Chewing areca nut (betel nut) increases the risk of oral cancer?	Strongly agree	152 (30.4%)	66.727	0.000*
		Agree	216 (43.2%)		
		Neither agree nor disagree	89 (16.1%)		
		Disagree	41 (8.2%)		
		Strongly disagree	10 (2.0%)		
9.	Do you have the habit of areca nut (betel nut) chewing?	No	396 (79.2%)	15.765	0.107
		Yes, I chew only areca nuts.	94 (18.9%)		
		Yes, I chew areca nuts along with tobacco.	10 (1.9%)		
10.	Are you aware of e-cigarettes (vaping/vapor products/electronic nicotine delivery systems)?	Yes	382 (76.4%)	4.139	0.941
		No	118 (23.6%)		
11.	The use of e-cigarettes affects oral health.	Yes	299 (59.8%)	1.394	0.999
		No	201 (40.2%)		
12.	Do you think that e-cigarettes will not cause staining of teeth and gum diseases?	Strongly agree	76 (15.2%)	24.390	0.041*
		Agree	141 (28.2%)		
		Neither agree nor disagree	167 (33.3%)		
		Disagree	87 (17.4%)		
		Strongly disagree	29 (5.9%)		
13.	Do you believe that e-cigarettes do not increase the risk of oral cancer?	Strongly agree	76 (15.8%)	14.967	0.243
		Agree	137 (27.5%)		
		Neither agree nor disagree	148 (29.7%)		
		Disagree	107 (21.5%)		
		Strongly disagree	29 (5.8%)		
14.	Do you feel e-cigarettes (vaping) are:	Absolutely healthy and safe	114 (22.8%)	8.296	0.217
		Less harmful than tobacco cigarettes but it is still not safe	97 (19.3%)		
		Equally harmful as tobacco cigarettes	249 (49.9%)		
		More harmful than tobacco cigarettes	40 (8.0%)		
15.	Do you use e-cigarettes?	No	420 (84.0%)	9.930	0.042*
		Yes, instead of tobacco cigarettes	19 (3.9%)		
		Yes, along with tobacco cigarettes	61 (12.1%)		
16.	Frequency of your habit of chewing smokeless tobacco/areca nut (betel nut)/using e-cigarette	One to three times a day	75 (15.0%)	21.816	0.001*
		Three to five times in a day	51 (10.2%)		
		Occasionally	83 (16.7%)		
		Not applicable	291 (58.1%)		
17.	At what age did you first begin chewing smokeless tobacco/areca nut/using e-cigarettes?	Less than 18 years	69 (13.7%)	42.294	0.000*
		18-30 years	9 (1.9%)		
		Above 30 years	47 (9.4%)		
		I am not sure	84 (16.9%)		
		Not applicable	291 (58.1%)		
18.	What made you/gave you the idea to start the habit?	Picked it up from my family member	53 (10.6%)	34.730	0.000*
		Picked it up from my friends	117 (23.4%)		
		TV commercials/social media	29 (5.8%)		
		No specific reason, I just started the habit	4 (0.8%)		
		Not applicable	297 (59.4%)		
19.	Reason to use e-cigarettes/vaping	Less harmful than smoking	79 (15.8%)	46.668	0.000*
		Less harmful to people around me	46 (9.2%)		
		Available in different flavors	9 (1.8%)		
		Helps to quit smoking cigarettes	21 (4.2%)		
		It does not smell, unlike smoking	16 (3.2)%		
		For style/fun	31 (6.2%)		
		If none of the above, state your reason…………	1 (0.2%)		
		Not applicable	297 (59.4%)		
20.	Do you want to quit the habit that you have currently (smokeless tobacco/areca nut/e-cigarettes)?	Yes, I am trying.	139 (27.8%)	27.075	0.001*
		Yes, I have tried but could not quit.	63 (12.6%)		
		No, I do not want to quit.	1 (0.2%)		
		Not applicable	297 (59.4%)		

## Discussion

Multiple studies have delved into the effects of smokeless tobacco, areca nut, and e-cigarette use on oral health among university students, revealing fascinating insights. Despite a commendable awareness of the dangers of smokeless tobacco and areca nut, a startling misconception persists about e-cigarettes, with many students mistakenly believing they are a healthier alternative to traditional cigarettes.

Smokeless tobacco usage and awareness

Understanding the impact of smokeless tobacco on oral health among university students reveals a complex picture of awareness and knowledge gaps. In our study, 427 (85.5%) participants recognized that chewing smokeless tobacco leads to poor oral health, reflecting a strong awareness similar to the 99.3% recognition reported [6]. However, when delving deeper into specific risks, the picture becomes less clear.

For instance, 198 (39.7%) of our respondents strongly agreed that smokeless tobacco leads to staining of teeth and gum diseases, and 196 (39.1%) strongly agreed that it increases the risk of oral cancer. This is in line with a previous study, which reported 78% awareness of the cancer risks associated with tobacco chewing, but only 27% awareness of premalignant lesions. This highlights a significant gap in comprehensive knowledge, despite high general awareness [4].

Further emphasizing the behavioral aspect, it found that 62% of users reported frequent use shortly after waking [13]. This aligns with our findings, where 6 (11.1%) of participants reported habitual use of smokeless tobacco. These insights underline the necessity for targeted interventions addressing both awareness and addiction.

In another study, despite high awareness post-workshop (98.58% acknowledging oral cancer as the most common cancer among Sri Lankan males), specific knowledge gaps persisted [17]. For instance, only 33.9% knew that smokeless tobacco products are banned in Sri Lanka, and awareness of cessation methods remained low (51.9% for counseling concepts). This highlights the need for ongoing education beyond basic awareness to include regulatory knowledge and cessation support.

Areca nut (betel nut) usage and awareness

Areca nut is the fourth most addictive substance in the world following tobacco, alcohol, and caffeine [3]. India is said to be the highest producer of areca nut in the world and it is evident that there exists a direct relation between the production and consumption of areca nut. India is the largest consumer of areca nuts, followed by China and Myanmar. There is a high prevalence of oral leukoplakia and OSMF in the Indian Subcontinent due to the practice of areca nut consumption.

An article, by Prashant Kumar Singh et al [9], reported that about 23.9% mature populace indulge in areca nut usage, that is, approximately 223.79 million people in India; most users consume areca nut with tobacco

The awareness of the dangers associated with areca nut use is generally high, but misconceptions remain prevalent. In our study, 411(82.1%) knew that areca nuts are present in commercial products like pan parag and Manik Chand pan masala, and 373(74.6%) acknowledged that chewing areca nuts leads to poor oral health. However, only 169(33.8%) strongly agreed that it leads to staining of teeth and gum diseases, revealing significant gaps.

Comparative studies, previously published show similar trends [4,9]. It was found that only 6% believed areca nut causes teeth staining, while 64% thought it causes oral cancer [18]. Our findings, where 152(30.4%) strongly agreed that areca nut increases the risk of oral cancer, reflect these mixed levels of understanding.

Another study reported that 52% of participants began using such substances between ages 12-16 years [7]. In contrast, only 69(13.7%) of our participants started before 18, suggesting later initiation but underscoring the need for early education. Bridging the gap between awareness and behavioral change remains a crucial area for intervention.

E-Cigarette awareness and perception

The disparity in awareness and perception of e-cigarettes compared to traditional tobacco products is striking. In our study, 382(76.4%) of students were aware of e-cigarettes, slightly lower than the 88.4% previously reported [12]. Despite this awareness, misconceptions persist, with 114(22.8%) of our respondents believing e-cigarettes are healthy and safe. and also found that 55% of students believed e-cigarettes were less harmful, and 37.5% thought they were useful for quitting smoking. Similarly, our study revealed that 79(15.8%) of participants believed e-cigarettes to be less harmful than smoking, and 16(3.2%) thought they helped quit smoking. This indicates a critical gap in understanding the actual risks associated with e-cigarette use. More cessation centers and ongoing education to combat rising smokeless tobacco use among South Asians effectively [13].

A study conducted previously involving 5,163 participants, revealed that while 78.4% believed e-cigarettes are harmful to health, misconceptions about their use persist [19]. Notably, 24.3% considered e-cigarettes a better option for patients than tobacco products, despite evidence suggesting otherwise. This disparity underscores the need for targeted education to dispel misconceptions and promote informed decision-making.

However, there are significant gaps in knowledge and perceptions about e-cigarettes [20]. Among their participants, only 13% understood the harmful effects, while 16% acknowledged the addiction potential. Our findings, where 299 (59.8%) recognized e-cigarettes' impact on oral health and 137 (27.5%) believed they did not increase the risk of oral cancer, emphasizing the need for comprehensive educational campaigns

Behavioral influences and initiation

Family and peer influence significantly impact the initiation of these habits. In our study, 117 (23.4%) of respondents picked up e-cigarette use from friends and 53 (10.6%) from family. These findings align with a previous study, which highlighted early initiation trends, with 70% of boys and 80% of girls starting tobacco use before the age of 15 years [11].

The allure of e-cigarettes often stems from perceptions of reduced harm and the potential to aid in quitting smoking. It found that 36.8% of respondents turned to flavored e-cigarettes to quit or reduce smoking, while our study reported only 16 (3.2%) for the same reason [10]. This discrepancy highlights the need for accurate information dissemination.

Multiple studies by various authors highlight widespread e-cigarette use among students, unveiling diverse awareness and attitudes shaped by cultural and social dynamics. These findings stress the urgent need for comprehensive education on vaping's risks and benefits to navigate these complex influences effectively [20-26].

Despite the awareness of the harmful effects, practices of tobacco, areca nut, and e-cigarettes are still being embraced, and in our study, it is seen that 27.8% (139) participants were trying to quit and 12.6% (63) tried to quit but failed to do so and were addicted. This highlights the importance of strategies to help with quitting and addiction.

The future scope of the study is to shed light on the current awareness levels regarding the association between oral health and the consumption of tobacco, areca nut, and e-cigarettes among college students in Chennai City. By providing valuable insights into this aspect, our research can serve as a foundation for developing targeted oral health education programs and raising awareness about the harmful effects of these substances. Given that such detrimental habits often take root early during college life, equipping students with adequate knowledge about the ill effects of tobacco, areca nut, and e-cigarette use is crucial [21-23,27-29]. This proactive approach can help prevent the development of potentially malignant disorders, including oral cancer, as well as mitigate other general health problems associated with these substances [21,25,30]. By empowering college students with the necessary information and awareness, our study aims to contribute to the promotion of oral health and the prevention of harmful habits, ultimately fostering healthier lifestyles among the youth population in Chennai.

The limitations of this study include that although valuable insights on the perspectives of college students in and around Chennai, on smokeless tobacco, areca nut, and e-cigarette use, are obtained, its reliance on voluntary participation may introduce a potential bias, as participants who chose to respond may differ systematically from those who did not. The focus only on university students which may limit the applicability of findings to non-student populations or individuals from different age groups or educational backgrounds with distinct attitudes and practices and cross-sectional design should be considered when interpreting the results. Future research employing longitudinal designs will be helpful to assess changes in behaviors and perceptions over time and to enhance the robustness of findings.

## Conclusions

Moving forward, our findings call for concerted efforts across academia, civil society, government, industry, and local communities. Collaboratively, we can develop comprehensive educational campaigns that transcend mere awareness to instill lasting behavioral change. Empowering college students with accurate knowledge about the risks of tobacco, areca nut, and e-cigarettes and the various strategies to help with quitting and de-addiction is pivotal in promoting oral health and fostering healthier lifestyles.

By leveraging this collective effort, we aspire to bring about policies, tailor-made, especially for our young population, safeguarding the well-being of future generations, and ensuring they thrive in environments that prioritize health and well-being.

## Appendices

Appendix

**Table 2:** Survey questionnaire regarding tobacco, areca nut, and e-cigarette use on oral health.

I consent to participate:	
Yes	
No	
Name:	
Age (in years):	Gender:
E-mail address:	Course of study:
Region / Location:	Marital status:
1. Chewing smokeless tobacco (Gutkha, Khaini, Hans etc) leads to poor oral health.	
Yes	
No	
2. Do you think that chewing smokeless tobacco leads to staining of teeth and gum diseases?	
Strongly agree	
Agree	
Neither agree nor disagree	
Disagree	
Strongly disagree	
3. Do you think that chewing smokeless tobacco increases the risk of oral cancer?	
Strongly agree	
Agree	
Neither agree nor disagree	
Disagree	
Strongly disagree	
4. Do you have habit of chewing smokeless tobacco (gutkha, khaini, hans etc)?	
Yes	
No	
5. Areca nut (also called betel nut)is present in commercial products like pan parag, manikchand pan masala, ajantha supari etc.	
Yes	
No	
6. Chewing areca nut (betel nut) leads to poor oral health.	
Yes	
No	
7. Do you think that Chewing areca nut (betel nut) can lead to staining of teeth and gum diseases?	
Strongly agree	
Agree	
Neither agree nor disagree	
Disagree	
Strongly disagree	
8. Do you think that Chewing areca nut (betel nut) increases risk of oral cancer?	
Strongly agree	
Agree	
Neither agree nor disagree	
Disagree	
Strongly disagree	
9. Do you have the habit of areca nut (betel nut) chewing?	
No	
Yes, I chew only areca nut	
Yes, I chew areca nut along with tobacco	
10. Are you aware of e-cigarettes (vaping/vapor products/electronic nicotine delivery systems)?	
Yes	
No	
11. Use of e- cigarettes affects oral health.	
Yes	
No	
12. Do you think that E cigarettes will not cause staining of teeth and gum disease?	
Strongly agree	
Agree	
Neither agree nor disagree	
Disagree	
Strongly disagree	
13. Do you believe that E cigarettes do not increase risk of oral cancer?	
Strongly agree	
Agree	
Neither agree nor disagree	
Disagree	
Strongly disagree	
14. Do you feel e-cigarettes (vaping)is/are?	
Absolutely healthy and safe	
Less harmful than tobacco cigarettes but it is still not safe	
Equally harmful as tobacco cigarettes	
More harmful than tobacco cigarettes	
15. Do you use e-cigarette?	
No	
Yes, instead of tobacco cigarette	
Yes, along with tobacco cigarette	
16. Frequency of your habit of chewing smokeless tobacco / areca nut (betel nut) / using e-cigarette.	
1 to 3 times in a day	
3 to 5 times in a day	
Occasionally	
Not applicable	
17. At what age did you first begin chewing smokeless tobacco/areca nut/using e cigarettes?	
Less than 18 years	
18 to 30 years	
Above 30 years	
I am not sure	
Not applicable	
18. What made you/gave you the idea to start the habit?	
Picked it up from my family member	
Picked it up from my friends	
TV commercials/ social media	
No specific reason, I just started the habit	
Not applicable	
19. Reason to use e-cigarettes/vaping.	
Less harmful than smoking	
Less harmful to people around me	
Available in different flavours	
Helps to quit smoking cigarettes	
It does not smell, unlike smoking	
For style / fun	
If none of the above, state your reason…	
Not applicable	
20. Do you want to quit the habit that you have currently (smokeless tobacco/areca nut/ e cigarettes)?	
Yes, I am trying	
Yes, I have tried but could not quit	
No, I do not want to quit	
Not applicable	
